# Fabrication of Artificial Food Bolus for Evaluation of Swallowing

**DOI:** 10.1371/journal.pone.0168378

**Published:** 2016-12-15

**Authors:** Miyu Hosotsubo, Tetsuro Magota, Masahiko Egusa, Takuya Miyawaki, Takuya Matsumoto

**Affiliations:** 1 Center for the Special Needs Dentistry, Okayama University Hospital, Shikata-cho, Kita-ku, Okayama, Japan; 2 Department of Dental Anesthesiology, Okayama University Graduate School of Medicine, Dentistry and Pharmaceutical Sciences, Shikata-cho, Kita-ku, Okayama, Japan; 3 Department of Biomaterials, Okayama University Graduate School of Medicine, Dentistry and Pharmaceutical Sciences, Shikata-cho, Kita-ku, Okayama, Japan; Kyoto Daigaku, JAPAN

## Abstract

Simple and easy methods to evaluate swallowing are required because of the recently increased need of rehabilitation for dysphagia. "Artificial food bolus", but not "artificial food", would be a valuable tool for swallowing evaluation without considering the mastication effect which is altered according to the individual's oral condition. Thus, this study was carried out to fabricate artificial bolus resembling natural food bolus. The mechanical property and the volume change of food bolus in normal people were firstly investigated. Thirty healthy adults without dysphagia were selected and asked to chew four sample foods (rice cake, peanut, burdock, and gummy candy). The results indicated that Young’s modulus of bolus before swallowing was below 150 kPa. The bolus volume before swallowing was below 400 mm^3^. In addition, the saliva component ratio of each bolus was approximately 30wt%, and the average saliva viscosity of research participants was approximately 10 mPa•s. Based on the obtained data, artificial food bolus was designed and fabricated by using alginate hydrogel as a visco-elastic material and gelatin solution as a viscotic material with a ratio of 7:3 based on weight. Consequently, the swallowing time of fabricated artificial food bolus was measured among the same participants. The results indicated the participants swallowed fabricated food bolus with similar manner reflecting their mechanical property and volume. Thus, this artificial food bolus would be a promising tool for evaluation of swallowing.

## Introduction

Intake of foods and acquisition of nourishment are crucial in living animals. Additionally, food intake is related to one's quality of life (QOL). However, the number of patients who need rehabilitation for dysphagia or who need nursing care has recently increased in this aging era. In this context, it is crucial to evaluate swallowing in patients in order to provide them with an optimal rehabilitation menu or nursing foods [1].

Currently, swallowing is often evaluated based on physiological properties [2,3]. For example, videofluorography is used to visualize in real time swallowing process of an object in a patient, which indicates deformation (if any) of the pharynx and esophagus tissues. This is one of the most powerful tools for the evaluation of swallowing [4], however, the evaluation system is expensive and time-consuming. Additionally, oral sensation, chewing, and salivation are important to evaluate swallowing. For example, the chewing gum test and saxon test are used for quantification of salivation [5]. In this test, a patient chews folded sterile sponge for 2 min, and the secreted saliva amount is measured. Moreover, a tongue pressure device consisting of disposable probe and manometer has been developed to understand the roles of tongue during evaluation process. This device can accurately measure the pressure generated by the whole tongue [6,7]. However, these tests do not directly indicate the swallowing function of patients. Thus, simple, easy, and inexpensive methods to evaluate swallowing are required.

There are several swallowing tests based on the use of test foods with different ingredients [8] for the evaluation of swallowing. However, the evaluated results are difficult to discuss owing to the variety of food properties [9]. In addition, the mastication is necessary before the food swallowing. A major problem using these test food, involves the variation in the mastication styles and strength among elderly persons due to the intraoral environmental differences such as dental loss, muscle weakness, and salivation reduction. Therefore physicians often find it difficult to determine whether the problem is based on mastication or swallowing.

In this context, the importance of standardized test foods for evaluation of swallowing has been emphasized in these years. It is desirable to reproduce the property of ingredients before swallowing for the test foods. Therefore, the purpose of this study was to fabricate "artificial food bolus" which resembles real food bolus before swallowing.

To achieve this goal, the mechanical property and volume change of food during mastication in normal people were firstly investigated. Next, the artificial food bolus was designed and fabricated according to the obtained data. Finally, the fabricated artificial food bolus was submitted to analysis of whether the bolus is sufficient for resembling the natural swallowing process.

## Methods

### 1. Selection of participants

Thirty healthy adult volunteers (male/female: 18/12) participated in this study. The criteria for selection of the participants were based on their ability to swallow test ingredients. Firstly, all the participants were observed swallowing saliva more than 3 times during swallowing inspection. This is one of the major swallowing evaluation methods termed Repetitive Saliva Swallowing Test (RSST), which is a simple test to check the individual’s swallowing ability. Through this test, a person with doubtful dysphagia was excluded [10,11]. In this study, participants provide their written informed consent to participate. This study and this consent procedure were approved by the ethical committee in Okayama University (authorization number 682).

### 2. Property change of different ingredients with chewing

Four different ingredients (rice cake, peanut, burdock, and gummy candy) were selected as sample foods (1000 mm^3^). The Young’s modulus of each ingredient is indicated as follows: rice cake; 25 kPa, peanut; 12,000 kPa, burdock; 2,400 kPa, and gummy candy; 180 kPa. Test foods were chewed by research participants, and spitted out after chewing for 5–40 times.

**Fig 1:**
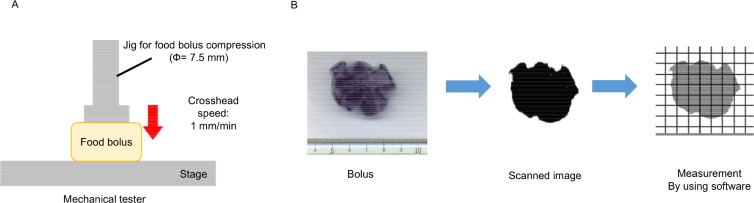
**Schematic diagram for measurement.** (A) Schematic diagram for measurement of bolus Young’ modulus. (B) Schematic diagram for measurement of bolus volume.

The chewed sample just before swallowing was also collected for measurement [12]. The state of "before swallowing" was determined when participants felt ready for swallowing. The Young’s modulus of collected bolus was measured by using a mechanical tester (EZ Test, Shimadzu, Japan). The sample was compressed by cylindrical jig attaching to the mechanical tester (Ф = 7.5 mm, crosshead speed: 1 mm/min) (Fig 1A). The force value obtained at 2 mm from the gel top after the compression was regarded as bolus Young’s modulus (JIS 7721).

The bolus volume in accordance with the chewing time was additionally measured. The bolus was molded with the prescribed height of 5 mm. The bottom image of adjusted bolus was acquired using an image scanner (Epson GT-F570, Japan). The area of the obtained image was analyzed using the image software (Image J, NIH, MD). The bolus volume was calculated from the obtained value (Fig 1B). The experiment was carried out in the air-conditioned room at 25°C.

The saliva contents in the sample bolus were measured by drying the collected samples. Further, the saliva viscosity of research participants was measured by using corn plate type viscometer (Toki industry, Japan) [13].

### 3. Fabrication of artificial food bolus

Generally, bolus is the mixture of food and saliva, and indicates viscoelastic properties. Therefore, alginate hydrogel as an elastic source and gelatin solution as a viscous source were selected to fabricate the artificial food bolus in this study. Sodium alginate is a polysaccharide-derivative of Phaeophyceae, which is an FDA-approved material, and is broadly used as a food additive and biomedical material [14]. Gelatin is denatured collagen derived from animal skins, which is also an FDA-approved material [15], and is broadly used as a food additive and biomedical material. Thus, it is well-known that both source materials are safe and cost-effective materials [16]. Sodium alginate solution (0.5-3wt%, 10 ml) was reacted with 20wt% of calcium chloride (10 ml). The mixed and reacted hydrogel was washed with distilled water for three times. The Young’s modulus of shaped gel (10 x 10 x 10 mm) was measured by using a mechanical tester (EZ Test, Shimadzu). Gelatin was dissolved in wormed double distilled water with 5-15wt% (Morinaga, Japan). The viscosity of each sample was measured by using the corn plate type viscometer (Toki industry). Finally, the mixture of alginate hydrogel and gelatin solution was prepared (7 to 3 in weight ratio, Table 1). The Young’s modulus of prepared materials was then measured by using a mechanical tester (EZ Test, Shimadzu).

**Table 1:** Composition of artificial food bolus.

Sample	Alginate hydrogel (mm^3^)	Gelatin (mm^3^)	Artificial food bolus (mm^3^)
1	560	240	800
2	420	180	600
3	280	120	400
4	140	60	200

### 4. Swallowing evaluation using the artificial food bolus

Artificial food boluses (100–800 mm^3^) with 47.3 kPa of Young’s modulus were prepared for the swallowing study of artificial food bolus. Also, three different volumes of artificial food boluses with different mechanical properties (4–200 kPa) were prepared for the swallowing test. The Young's modulus and volume used in this study were selected based on the results obtained through the study of section 2. Swallowing time of artificial food bolus was evaluated by measuring the time of laryngeal elevation of participants during swallowing.

### 5. Statistical analysis

Quantitative tests were conducted in quadruplicate, and mean values with standard deviations were then calculated. Statistical significance at the 1% level was evaluated using one-way analysis of variance (ANOVA) with Scheffe’s F test. All data points in the graphs represent the mean values and the error bars represent the standard deviations.

## Results

### 1. Properties change of ingredients with chewing

**Fig 2:**
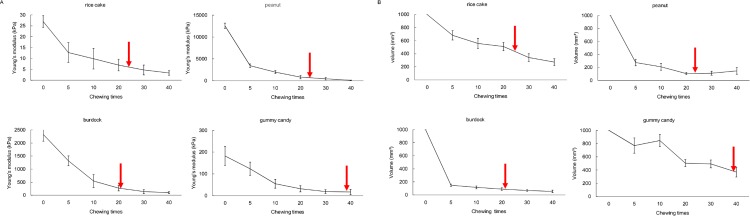
**Properties change of different ingredients with chewing.** (A) Alterations of Young's modulus according to the mastication time. All sample foods indicate a drastic decrease of Young's modulus within 10 times, and turn to the moderate decrease phase. Red arrow in graph represents the mean swallowing time of research participants. (B) Alterations of volume according to the mastication time. All sample foods indicate a volume decrease during mastication. Red arrow in graph represents the mean swallowing time of research participants.

Fig 2A indicates the Young’s modulus change of four test foods before chewing and just before swallowing. The Young’s modulus of rice cake was 25 kPa before chewing, and gradually decreased at a range of 1–6 kPa before swallowing. The mean chewing times until swallowing was 24.76 times. The Young’s modulus of peanut was 12,000 kPa before chewing and was 100–150 kPa before swallowing. The mean chewing times until swallowing was 23.2 times. The Young’s modulus of burdock was 2,400 kPa and became 80–100 kPa before swallowing. The mean chewing times until swallowing was 21.25 times. The Young’s modulus of gummy candy was 180 kPa, and became 10–20 kPa before swallowing. The mean chewing times until swallowing was 39.27 times. Bolus mechanical stiffness decreased with the increase of chewing times. Interestingly, the Young’s modulus of each food changed drastically within 10 times after the start of chewing and then changed their property moderately. Totally, the bolus Young’s modulus during swallowing was less than 150 kPa in all ingredients. In addition, swallowing was observed in the "moderate change period".

Fig 2B indicates the volume change of sample foods during mastication. A decrease of volume was observed in each food according to the chewing. The final volume before swallowing was at a range of 200–300 mm^3^ in the rice cake. For other foods, the values were 100–150 mm^3^, 100–150 mm^3^, and 300–400 mm^3^ for peanut, burdock, and gummy candy, respectively. Thus, all bolus volume values during swallowing were less than 400 mm^3^.

**Fig 3:**
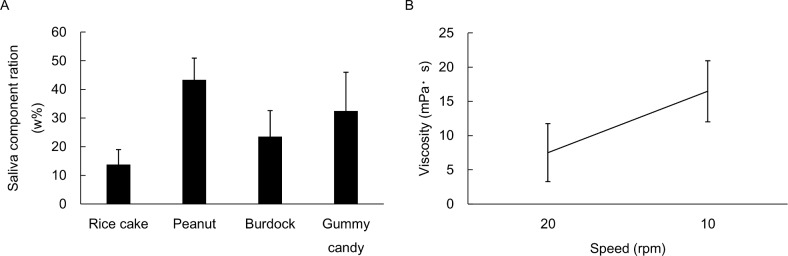
**Saliva viscosity and Weight ratio of saliva.** (A) Saliva viscosity of research participants measured by viscometer. (B) Weight ratio of saliva contained in the obtained bolus soon before swallowing.

The saliva viscosity of participants was in the range of 7.5–16.4 mPa•s, and the mean value was 10.0 mPa•s (Fig 3A). The saliva content varied according to the sample foods, and the contained rate was in the range of 13.8–43.4wt%. The mean value was approximately 30wt% (Fig 3B).

### 2. Fabrication of artificial food bolus

**Fig 4:**
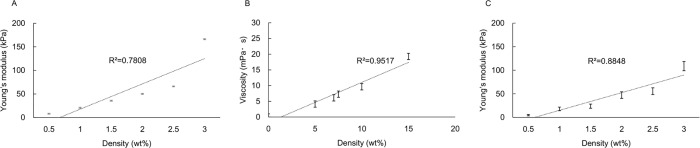
**Fabrication of artificial food bolus.** (A) Young's modulus of alginate hydrogel. The value increases according to the sodium alginate concentration. (B) Viscosity of gelatin solution. The value increases according to the gelatin concentration. (C) Young's modulus of artificial food bolus. The value mainly reflects the Young's modulus of alginate hydrogel.

The Young's modulus of alginate hydrogel increased depending on the sodium alginate concentration (Fig 4A). The viscosity of gelatin solution increased depending on the gelatin concentration (Fig 4B). Since the obtained results indicated that the average saliva contents in sample bolus was approximately 30wt%, the artificial food bolus was fabricated by the mixture of alginate hydrogel and gelatin solution with 7:3. The Young’s modulus of the obtained artificial food bolus indicated that the value was lower than that for the alginate hydrogel per se; however, it still reflects the sodium alginate concentration (Fig 4C).

### 3. Swallowing evaluation using artificial food bolus

**Fig 5:**
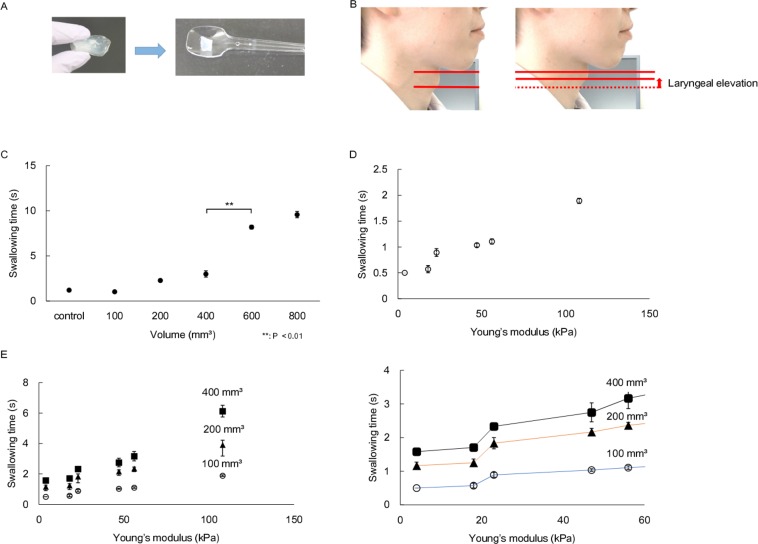
**Swallowing evaluation using artificial food bolus.** (A) The prepared artificial bolus for swallowing evaluation. The artificial food bolus was molded with the prescribed volume. (B) Laryngeal elevation (red arrow) time was measured as swallowing time. (C) The swallowing time of artificial bolus with different volume. The time increases linearly until the volume of 400 mm^3^, and then largely increases more than this volume (P **<0.01). (D) The swallowing time of artificial food bolus with different Young's modulus. The time increases linearly until the modulus below 110 kPa. Participants are unable to swallow the artificial bolus of 200 kPa without mastication. (E) The swallowing time of artificial food bolus (100–400 mm^3^) with different Young's modulus. The swallowing time of artificial bolus below 23.8 kPa indicates the significantly rapid swallowing.

To confirm that the fabricated artificial food bolus shows natural swallowing process similar to the normal food, the swallowing time of the fabricated artificial food bolus was measured (Fig 5A and 5B). Firstly, the swallowing time of the fabricated bolus of the same mechanical property with different volume was measured. The results indicated that the swallowing time was linearly increased according to the volume at a range of 100–400 mm^3^. Then, the swallowing time was drastically increased over 400 mm^3^ of artificial food bolus (Fig 5C). Next, the swallowing time of the fabricated bolus of the same volume with different mechanical properties was measured. The results indicated that swallowing time was linearly increased according to the mechanical property at a range of 4–100 kPa (Fig 5D). However, research participants were unable to swallow artificial food bolus of 200 kPa without mastication. This phenomenon was also confirmed in samples with different volumes (100–400 mm^3^). Interestingly, the swallowing time of samples less than 20 kPa in Young’s modulus showed significantly shorter time in each bolus volume (100–400 mm^3^)(Fig 5E).

## Discussion

The conventional swallowing evaluation method is expensive and complicated. Therefore, a cost-effective and simple method for this evaluation is desired. “Special foods for a patient with dysphagia” have been designed and applied for the evaluation of swallowing; however, such foods have not been developed solely for the swallowing test [17,18]. In addition, since the test foods need mastication before swallowing, it is difficult to evaluate only the swallowing function [19,20]. Thus, development of novel test materials that can be used specifically for the swallowing test is paid attention in these years. Therefore, "the artificial food bolus" was fabricated in attempt to resemble real food bolus before swallowing, and could be used for evaluation of swallowing function.

Initially, the change of Young’s modulus and volume of natural food bolus were investigated. All four sample foods led to the decrease of Young’s modulus during mastication and indicated two different decrease phases: "Rapid change period" and "Moderate change period". The "Rapid change" of Young’s modulus of bolus occurred within 10 times of mastication, and then Young’s modulus of the food bolus changed slightly, that is, "Moderate change period". All food boluses were swallowed in this "Moderate change period". Thus, it seemed that one does not swallow food bolus until the mechanical properties of bolus settle down. Previous report indicated that the activity rhythm of masticatory muscles become smaller before swallowing [21]. Thus, the stability of properties change of food bolus would be crucial for swallowing.

In addition, the volume of natural food bolus decreased according to the mastication. This decrease of bolus volume seemed to be caused by "intraoral remaining" and "stage II transport" [22]. Peanut and burdock did not tend to form one aggregated food bolus because of their poor adhesion property, but facilitated to stay in occlusal surface or interdentium [23]. Rice cake and gummy candy have high cohesiveness, and tend to form aggregated food bolus. Moreover, some portion of food bolus is dissolved or becomes sol-like, and moves into the pharynx as stage II transport [24]. The swallowing process is commonly divided into oral, pharyngeal, and esophageal stages according to the location of the bolus. The movement of the food to the oropharynx in the oral cavity differs between eating solid food and drinking liquid. Therefore, the solution-like phase of the bolus seems to be easily carried to the oropharynx [25,26]. Consequently, all food bolus before swallowing indicated less than 150 kPa in Young’s modulus, and less than 400 mm^3^ in volume.

Based on the obtained data, the artificial food bolus was designed and fabricated using alginate hydrogel and gelatin solution. Alginate hydrogel shows elastic property, and gelatin solution shows viscotic property [27,28]. Since food bolus shows visco-elastic properties, the mixture of these materials were selected for use in this study. Each single material showed higher elasticity and viscosity according to the concentration of source material. Since the obtained result indicated that the mean value of saliva contents in food bolus was approximately 30wt%, the artificial food bolus fabrication was conducted according to this rate.

To confirm the usefulness of the fabricated artificial food bolus, the swallowing evaluation was performed using the same research participants. Previous studies indicated that mastication affects the swallowing timing [29]. Therefore, participants were asked to swallow the artificial food bolus without mastication. The swallowing time of the prepared artificial food bolus increased linearly when research participants swallow the artificial food bolus of below 400 mm^3^ in volume and below 100 kPa in Young’s modulus. Research participants took time or were unable to swallow the artificial food bolus more than 400 mm^3^ in volume and more than 200 kPa in Young’s modulus. In addition, swallowing time of artificial bolus below 23 kPa of Young’s modulus showed significantly short swallowing time. This is probably because participants recognized this bolus as sol phase. Previous studies revealed that solution or sol type foods indicate less than 20 kPa in Young’s modulus [30]. Altogether, it was confirmed that the fabricated artificial food bolus reflects one's normal swallowing.

This artificial food bolus is specific for evaluation of swallowing. The physicians need to understand and estimate the swallowing functions of patients easily and comparably. For nursing care, a caretaker or assistant for rehabilitation needs to know the optimal size and stiffness of food for patients. The fabricated artificial food bolus would be a promising tool for these purposes. The mechanical property and volume of food bolus were mainly focused in this study, however, there are other important bolus properties such as tribology for the special mouth feel. These additional properties should be discussed in the next step of this work [31].
